# Healthcare Burden Associated with Malnutrition Diagnoses in Hospitalized Children with Critical Illnesses

**DOI:** 10.3390/nu15133011

**Published:** 2023-07-01

**Authors:** Julie Khlevner, Kelly Naranjo, Christine Hoyer, Angela S. Carullo, Kirk W. Kerr, Barbara Marriage

**Affiliations:** 1 Division of Pediatric Gastroenterology, Hepatology and Nutrition, Columbia University Vagelos College of Physicians and Surgeons, New York, NY 10032, USA; 2 Department of Biology, New York University, New York, NY 10032, USA; kellyvictoria.naranjo@gmail.com; 3 New York Presbyterian Morgan Stanley Children’s Hospital, New York, NY 10032, USA; chh9095@nyp.org (C.H.); anc9214@nyp.org (A.S.C.); 4 Abbott Laboratories, Columbus, OH 43023, USA; kirk.kerr@abbott.com (K.W.K.); bmarriage@outlook.com (B.M.)

**Keywords:** malnutrition, pediatrics, nutrition screening, nutritional interventions, undernutrition, clinical outcomes, mid-upper arm circumference

## Abstract

Our primary study objectives were to (i) determine the proportion of children admitted to the Pediatric Intensive Care Unit (PICU) with malnutrition diagnoses, (ii) compare healthcare utilization by malnourished and non-malnourished PICU patients, and (iii) examine the impact of implementing malnutrition screening and coding practices at a major academic urban tertiary care medical center. Using patient records, we conducted a retrospective analysis of 4106 children admitted to the PICU for severe illnesses between 2011 and 2019. Patients were identified as malnourished if records showed an ICD-9 or ICD-10 code for malnutrition. We compared malnourished and non-malnourished patients by age, admitting diagnoses, number of comorbid conditions, and clinical outcomes (length of stay, hospital readmission). About 1 of every 5 PICU-admitted patients (783/4106) had a malnutrition diagnosis. Patients with malnutrition were younger (mean age 6.2 vs. 6.9 years, *p* < 0.01) and had more comorbid conditions (14.3 vs. 7.9, *p* < 0.01) than those without. Malnourished patients had longer hospital stays (26.1 vs. 10.0 days, *p* < 0.01) and higher 30-day readmission rates (10% vs. 7%, *p* = 0.03). Implementation of malnutrition screening and coding practices was associated with an increase in malnutrition diagnosis. In this study of children admitted to the PICU, malnourished patients had more comorbid diagnoses and used more healthcare resources (prolonged hospitalizations and higher 30-day readmission rates), leading to higher healthcare costs. Such findings underscore the need for policies, training, and programs emphasizing identification and treatment of malnutrition at hospitals caring for critically ill children.

## 1. Introduction

Patients admitted to the pediatric intensive care unit (PICU) may have either acute illness or acute exacerbations of complex chronic conditions. Common reasons for admission to the PICU include respiratory disease, cardiac disease, and neurologic disorders [1,2]. Children with special needs comprise as many as 38% of PICU admissions [1]. Severe sepsis and septic shock are also common to patients in PICUs, with a prevalence of more than 8% worldwide and a mortality rate exceeding 24% [1]. A 2015 United States (US) study estimated annual PICU admissions at more than 2 million cases [3]. Nearly 40% of these patients had a length of stay (LOS) greater than 7 days, and more than 40% required mechanical ventilation [1]. In 2015, median per-patient PICU charges in the US were about $50,000, which corresponded to a total of nearly $25 billion overall [3].

In children, malnutrition (or undernutrition) can either contribute to disease vulnerability or may be caused by the disease itself [4]. For example, gastrointestinal, heart, respiratory, and kidney defects; cerebral palsy; cystic fibrosis; short bowel syndrome; solid tumor cancers; and third-degree burns put infants and children at high risk of malnutrition [5]. On the other hand, some children receive foods with low nutritional value because their family does not follow a diverse diet or because their family cannot afford nutritious foods or lacks access. Approximately 10% of US households were food insecure in 2022 [6]. Children with malnutrition are more susceptible to infections, which also increases risk for mortality [7]. In-hospital recognition and treatment of disease-associated malnutrition is, thus, an imperative component of care for all pediatric patients, especially for those children who are critically ill.

Our study involved critically ill pediatric patients admitted to an urban academic medical center; our primary objectives were to (i) determine the proportion of children admitted to the PICU with malnutrition diagnoses, (ii) compare healthcare utilization by malnourished and non-malnourished PICU patients, and (iii) examine the impact of implementing malnutrition screening and coding practices. Optimal management of hospitalized infants and children who require critical care is essential and can be lifesaving. We posit that attention to nutritional needs is an important component of PICU care. Hospitals across the nation thus need public health standards to ensure access, trained staff, and guidelines for nutritional assessment and care of hospitalized children, especially those in the PICU.

## 2. Materials and Methods

In 2013, New York-Presbyterian Morgan Stanley Children’s Hospital (NYP-MSCH) implemented a program to improve malnutrition screening and coding with a goal to identify pediatric malnutrition in a timely manner and standardize the threshold for intervention. Patients at NYP-MSCH were diagnosed as malnourished when their weight for length or BMI were z-scores of −1 (mild malnutrition), −2 (moderate malnutrition), or −3 (severe malnutrition), or when the patients had an unplanned decline in these z-scores of 1, 2, or 3 standard deviations. In 2015, the mid-upper arm circumference (MUAC) measurement tool was introduced as a second criterion to screen for malnutrition and prompt further assessment for malnutrition diagnosis. The MUAC measurement requires simple equipment (paper tape) and is easy to perform, even on medically complex patients. It is a valuable screening tool to assess malnutrition risk in children 3 months through 18 years of age. In children aged 3–60 months, per The World Health Organization, MUAC of less than 11.5 cm meets the criterion for severe malnutrition [8,9]. Screening is conducted using a flexible tape measure wrapped around the patient’s mid-upper arm to measure the arm circumference for comparison to established Academy of Nutrition and American Society for Parenteral and Enteral Nutrition criteria [10]. Based on the available recommendations, an MUAC z-score between −1 and −1.9 indicates mild malnutrition, z-score between −2 and −2.9 indicates moderate malnutrition, and z-score −3 or less indicates severe malnutrition. NYP-MSCH Registered Dietitians were trained on the use of MUAC tape measure utilizing the Nutrition-focused Physical Exam presentation in 2013–2015 and demonstrated techniques during team training. Documentation of MUAC measurements were added to the malnutrition diagnoses criteria in 2015.

### 2.1. Patient Population

This retrospective study used electronic medical records (EMR) from a 200-bed pediatric hospital in a tertiary care academic medical center in an urban setting. This study included children admitted to the 41-bed Pediatric Intensive Care Unit (PICU) from 2011 through 2019 (*n* = 8725). From the EMR, a subset of non-neo-natal PICU patients who had received a coded diagnosis of malnutrition (CDM) was identified based on ICD-9 (262, 263.0, 263.1, 783.22) or ICD-10 codes (E43, E44.0, E44.1, E46, Z68.51, Z71.3), but was not otherwise limited by patient diagnosis or procedure.

### 2.2. Measures and Comparisons

For demographic and medical features, race, sex, age, and number of comorbid health conditions were obtained. As a measure of illness severity, we calculated the Elixhauser comorbidity index from diagnostic codes in hospital records [11]. The Elixhauser index predicts the likelihood of in-hospital mortality and 30-day hospital readmission for patients based on the presence of any of 30 comorbidities in patients’ health records. Likelihood of in-hospital mortality and 30-day readmission are related to patient severity of illness. For healthcare utilization, we noted patients’ hospital LOS and 30-day hospital readmissions from electronic medical records of the hospital. The cost of a PICU stay was estimated to be $5799 using daily PICU cost data from a study of an academic PICU in the US and was inflation-adjusted to 2022 dollars using the CPI for Medical Care [12,13].

### 2.3. Statistical Analyses

Patient characteristics for malnourished and non-malnourished patients as well as patients from the pre-2015 and post-2015 cohort were compared using Wilcoxon and chi-squared tests. Patients from the pre-2015 and post-2015 cohorts were matched on age, sex, race, and Elixhauser index to control for observed differences between the cohorts. Length of stay and 30-day hospital readmission were compared in the matched sample using Wilcoxon and t-tests, respectively. Since a simple mean or median comparison, even with a matched sample, does not account for the impact of other covariates on outcomes, ordinary least squares (OLS) regression of hospital LOS on patient characteristics and indicators for the period after implementation of the hospital program emphasizing malnutrition diagnosis and care were performed.

## 3. Results

### 3.1. Patient Demographics

Based on medical records of children admitted to the PICU of our academic medical center from 2011 through 2019 (*n* = 4106), we found that 783 (19.1%) had CDM. The mean age was 6.74 years (±6.12 standard deviation), and 52% of patients were males (Table 1).

### 3.2. Illness Severity

Minor demographic and notable clinical differences between malnourished and not malnourished PICU patients were identified (Table 2 and Table 3). Malnourished PICU patients were slightly younger (6.17 yrs. vs. 6.88, *p* < 0.01) and more often male (55.6% vs. 51.2%). Malnourished PICU patients had nearly twice as many clinical diagnoses as non-malnourished patients, indicating these children were more severely ill (Table 3). Additionally, mean scores on the Elixhauser comorbidity index risk of 30-day all-cause hospital readmission and for risk of in-hospital mortality were significantly higher in children with malnutrition (Table 3).

### 3.3. Identifying Malnutrition Risk by Use of Mid-Upper-Arm Circumference (MUAC) Measure

**Figure 1:**
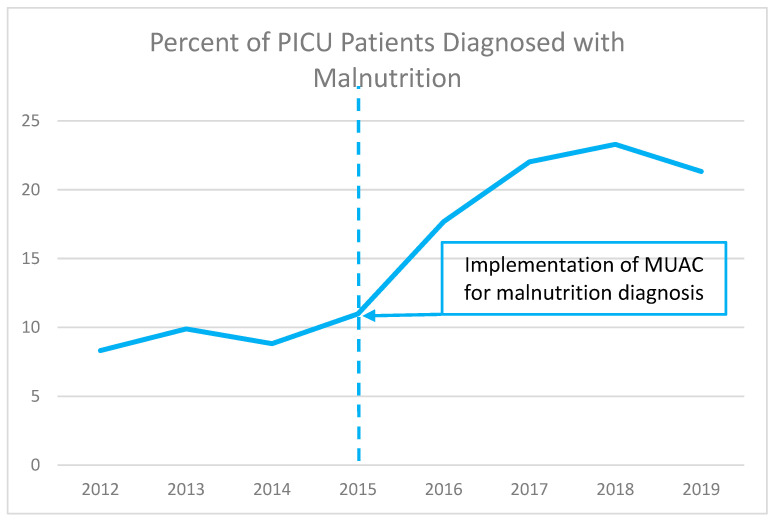
Percentage of admitted patients identified as malnourished before and after implementation of MUAC measurement.

Figure 1 shows the percentage of admitted PICU patients who were identified as malnourished from 2012–2019. Following implementation of MUAC-based detection of malnutrition in 2015, we found more than 20% of PICU patients were reported as malnourished by 2017, and this level was consistently observed for the duration of our study. This finding suggests that MUAC is a practical tool for identifying patients with malnutrition in the PICU.

### 3.4. Impact of Malnutrition on Healthcare Use and Costs of Care

In the present analysis, the data show that patients with diagnosed malnutrition experienced mean PICU stays that were 2.6 times or 16 days longer than those of non-malnourished patients (26.11 vs. 10.0, *p* < 0.01) (Table 4). This is likely related to patient complexity and the presence of multiple comorbid conditions. Additionally, a higher number of PICU patients with malnutrition were readmitted within 30 days than non-malnourished PICU patients (0.10 vs. 0.07, *p* = 0.03).

Previous research has estimated the average cost of a day of PICU care at $5799 for medical patients (values inflation-adjusted to 2022 dollars) [12]. Applying the average cost per day for medical patients to the average length of stay yields estimated total costs of $151,401 for malnourished patients and $57,986 for patients without malnutrition—a cost difference of $93,415 per patient.

### 3.5. Regression Analysis of Factors Affecting Patient Length of Stay

Patient characteristics in the periods before (pre-2015) and after (post-2015) implementation of malnutrition coding are presented in Table 5. Patients have similar distributions of race and sex, but post-2015 patients are significantly older and have a significantly higher Elixhauser mortality index. These differences in patient characteristics could result in differences in patient outcomes, so patients are matched on race, age, sex, and Elixhauser indexes to obtain pre- and post-2015 samples with similar characteristics. Matching on patient characteristics results in a smaller sample, but there are not significant differences in characteristics between the pre- and post-2015 cohorts (Table 5) after matching.

The regression analysis shows a strong positive association between CDM and longer LOS (Table 6). The negative coefficient for patients admitted in 2015 or later indicates that they had significantly shorter lengths of stay than did those admitted pre-2015. The negative coefficient on age indicates that older patients had shorter LOS. Malnutrition was again associated with a longer length of stay. Coefficient estimates indicated that a patient diagnosed with malnutrition would have a stay 101% longer than a patient not diagnosed with malnutrition. This finding indicates that a malnutrition diagnosis was associated with 10.9 additional PICU days compared to patients not diagnosed with malnutrition, and an incremental cost of $63,134.

## 4. Discussion

### 4.1. Overview of Findings

Diagnosing pediatric malnutrition is imperative to establish the true prevalence of the disorder and provide adequate treatment and education. Additionally, identifying patients with malnutrition and providing them with appropriate care can improve patient outcomes and reduce unnecessary hospital readmissions. In our retrospective study at a US academic tertiary medical center, almost 1 of every 5 PICU-admitted patients (783/4106) had a CDM. Patients with malnutrition were younger (mean age 6.2 vs. 6.9 years, *p* < 0.01) and had more comorbid conditions (14.3 vs. 7.9, *p* < 0.01) than those without malnutrition. Patients with CDM had 2.6-times longer hospital stays (26.1 vs. 10.0 days, *p* < 0.01) and higher 30-day readmission rates (10% vs. 7%, *p* < 0.01). After implementation of the malnutrition screening protocol, there was a decline in length of stay noted in all groups. The analysis controlled for patient characteristics and observable differences between groups. Estimates showed that longer PICU stays for patients diagnosed with malnutrition cost over $60,000 per patient stay.

### 4.2. Pediatric Nutrition Screening and Assessment in Perspective

For hospitalized patients, malnutrition is associated with poor health outcomes and greater risk for hospital readmission [14,15]. Malnutrition is common and is associated with risk for adverse outcomes in hospitalized children who are fragile or have complex medical conditions [16,17]. Our study findings show that undernourished children had longer PICU hospitalization stays and were more likely to be readmitted, which led to greater healthcare costs. Recognizing and accurately coding for malnutrition in medical records of critically ill, hospitalized children will reveal the true prevalence of malnutrition and can promote malnutrition treatment.

In our study sample, 19.1% of patients had a CDM, as compared to previously published data of 1.3% in hospitalized children, though these earlier studies were not limited to PICU patients [18,19]. Similar to a previous study of LOS in children with malnutrition, our findings for PICU patients showed LOS for patients with malnutrition was 2.6 times longer than for those without malnutrition [18]. One potential driver for the high incidence of malnutrition in our cohort is likely the ICU setting, where more severely ill patients are hospitalized and who are more likely to be malnourished. Additionally, previous studies did not look specifically at diagnoses made using the MUAC tool, which, by simplifying screening and identification, should make diagnosis easier and, to some extent, more common.

Given the association between malnutrition and poor patient outcomes, as well as increased costs of hospital care, providers now have a clear rationale to expedite recognition and treatment of malnutrition. Specific goals to help address malnutrition in hospitalized children are to identify the best tools to (i) screen for malnutrition risk, and (ii) assess for diagnosis of malnutrition and determine its severity [20,21]. In spite of an early call for malnutrition screening tools [22], there is still no clear agreement on which tools are best for the inpatient setting. In fact, at least ten tools are available to screen and assess hospitalized children for malnutrition risk [20,22,23,24,25,26]. We posit that simplicity is of great importance when implementing a malnutrition screening protocol, and our experience indicated that MUAC was easy and practical to use. Due to its ease of use, the tool can be used post-hospitalization by community health workers as needs arise and facilitate tracking of children who are not typically seen within a particular medical center (e.g., recent immigrants or those who have moved to an area), to easily prevent these patients from being overlooked.

MUAC is recognized as validated nutrition screening tool [27,28], an accurate predictor of malnutrition and wasting among children [29,30], and is typically used for infants and children from 3 months to 18 years of age [31]. Additionally, MUAC is a good predictor of mortality, and, in many studies, MUAC predicted death in children better than any other anthropometric indicator [32,33,34,35]. However, this study did not examine the use of MUAC in predicting mortality or readmission. MUAC is less affected than weight and height indices by localized accumulation of fluid and is more accurate, regardless of fluid status, body habitus, or multiple comorbidities/conditions. An advantage of the MUAC for major clinical outcomes is in standardization of coding practices to track and correlate outcomes to other anthropometrics used clinically. However, Stephens noted that MUAC has limitations, including identification of appropriate cutoff points by age and sex [30].

Two additional points are important to the clinical implications of our study findings. First, once malnutrition is diagnosed, nutritional interventions should be in place. Such interventions may include provision of micro- and macronutrients [36,37]. Second, routine screening for malnutrition should be implemented across all pediatric care within the community (primary care, other specialty care clinics) to improve early detection and immediate intervention [21].

In this study of children admitted to the PICU, malnourished patients had more comorbid diagnoses, prolonged hospitalizations, and increased 30-day readmission rates, all leading to higher healthcare utilization. Implementation of screening and coding for diagnosis of malnutrition in our center was associated with a reduction in hospital LOS. We estimated that the costs of PICU care for a malnourished child could be as much as $100,000 more per hospitalization than for a child without malnutrition. Such findings underscore the need to build hospital policies, train staff, and implement programs for malnutrition identification and treatment at hospital sites caring for critically ill children.

### 4.3. Study Limitations

This study has limitations related to its conduct and implications. The study focused on the impact of implementing a diagnostic process on the number of diagnoses, and the impact of malnutrition on patient outcomes. However, changes in patient care due to diagnosis were not examined and should be the topic of future research. Thus, the implications of the study relate to the ability of the process to identify malnourished patients and describe the impact of malnutrition on patient outcomes. Additionally, the study used a convenience sample of available patient data between 2011 and 2019, so no power analysis was conducted to determine the relevance and generalizability of our statistical results. Our sample also lacked information on some patient characteristics that may have impacted malnutrition incidence, as well as patient comorbidities and outcomes. Socioeconomic status, race, and ethnicity are correlated with food insecurity and other drivers of malnutrition and patient outcomes, but were not available in our dataset.

Additionally, this study used the Elixhauser comorbidity index to control for patient severity. This index, originally developed for adult inpatients, is commonly used to measure comorbidities and patient severity of illness and is useful because it was designed for use in administrative data such as those used in this study. However, some of the comorbidities included in the index, such as congestive heart failure and chronic pulmonary disease, may not be as prevalent or predictive in a pediatric population. Although not designed for pediatric populations, it has been analyzed in pediatric populations and found to perform similar to other comorbidity measures [38]. Comparable measures of pediatric comorbidities are still under development [2].

## 5. Conclusions

In this study of children admitted to the PICU, malnourished patients had more comorbid diagnoses, prolonged hospitalizations, and increased 30-day readmission rates, all leading to higher healthcare utilization. Implementation of screening and coding for diagnosis of malnutrition in our center was associated with a reduction in hospital LOS. We estimated that costs of PICU care for a malnourished child could be as much as $100,000 more per hospitalization than for a child without malnutrition. Such findings underscore the need to build hospital policies, train staff, and implement programs for malnutrition identification and treatment at hospital sites caring for critically ill children.

## Figures and Tables

**Table 1:** Demographics of children admitted to PICU 2011–2019.

Variable	
Age (years), Median (IQR)	5 (1–12)
Male, % of total	52%
White, % of total	75.8%
Black, % of total	17.1%
Asia/Pacific Islander, % of total	6.4%
Other, % of total	0.7%

IQR—Interquartile range (25th Percentile–75th Percentile).

**Table 2:** Demographics of patients with and without malnutrition diagnosis.

Variable	With Malnutrition Diagnosis (*n* = 783)	No Malnutrition Diagnosis (*n* = 3323)	*p*-Value
Age, Median (IQR)	3 (0–12)	5 (1–12)	<0.01
Male, *n* (%)	435 (55.6%)	1700 (51.2%)	0.03
White, *n* (%)	581 (74.2%)	2532 (76.2%)	0.23
Black, *n* (%)	134 (17.1%)	568 (17.1%)	
Asia/Pacific Islander, *n* (%)	63 (8.1%)	201 (6.1%)	
Other, *n* (%)	5 (0.6%)	22 (0.7%)	

IQR—Interquartile range (25th Percentile–75th Percentile).

**Table 3:** Illness severity and number of secondary diagnoses in PICU patients with and without a coded diagnosis of malnutrition.

	With Malnutrition Diagnosis (CDM) *n* = 783	No Malnutrition Diagnosis *n* = 3323	*p*-Value
Secondary diagnoses, Mean (S.D.)	13 (8–18)	6 (3–11)	<0.01
Elixhauser readmission risk index, Median (IQR)	0 (0–4)	0 (0–4)	<0.01
Elixhauser mortality risk index, Median (IQR)	0 (0–2)	0 (0–2)	<0.01

IQR—Interquartile range (25th Percentile–75th Percentile).

**Table 4:** Healthcare use and estimated excess costs of care in malnourished versus non-malnourished PICU patients.

	With Malnutrition Diagnosis (CDM) *n* = 783	No Malnutrition Diagnosis *n* = 3323	*p*-Value
Length of stay (days), Median (IQR)	13 (6–28)	5 (3–10)	<0.01
30-day readmission (occurrence), Mean (S.D.)	0.10 (0.29)	0.07 (0.26)	0.03

IQR—Interquartile range (25th Percentile–75th Percentile); S.D., standard deviation.

**Table 5:** Patient characteristics before and after implementation of malnutrition coding.

	**Before Matching**			**After Matching**		
	Pre-2015 Patients (*n* = 304)	Post-2015 Patients (*n* = 3802)	*p*-Value	Pre-2015 Patients (*n* = 304)	Post-2015 Patients (*n* = 304)	*p*-Value
Asia/Pacific Islander, %	5.6%	6.5%	0.32	5.6%	5.6%	0.95
Black, %	13.8%	17.4%		13.8%	13.8%	
White, %	80.6%	75.4%		80.6%	80.6%	
Other races, %	0%	0.7%		0%	0%	
Male, %	50.0%	52.2%	0.47	50.0%	50.0%	0.99
Female, %	50.0%	47.8%		50.0%	50.0%	
Age, Mean (S.D.)	4.95 (4.29)	6.89 (6.22)	<0.01	4.95 (4.29)	5.03 (4.53)	0.83
Elixhauser Readmission Index, Mean (S.D.)	1.50 (2.39)	1.77 (3.12)	0.06	1.50 (2.39)	1.34 (2.29)	0.38
Elixhauser Mortality Index–Mean (S.D.)	1.32 (3.67)	1.84 (5.60)	0.02	1.32 (3.67)	1.00 (3.60)	0.28

S.D., standard deviation.

**Table 6:** Regression analysis of length of stay (*n* = 608).

Dependent Variable = ln(Length of Stay)	
Variable	Coefficient (Standard Error)
Diagnosed malnutrition	1.01 ** (0.21)
Admitted 2015 or later	−0.35 ** (0.09)
Admitted 2015 or later + diagnosed malnutrition	0.06 (0.27)
Female	0.02 (0.09)
Age	−0.05 ** (0.01)
Asia/Pacific Islander	0.23 (0.19)
Black	0.01 (0.13)
Elixhauser Readmission	0.03 (0.02)
Elixhauser Mortality	−0.01 (0.01)

**—significant at α ≤ 0.01.

## Data Availability

Data available upon request from the corresponding author. The data are not publicly available due to privacy concerns.
